# Pharmaceutical Innovation and Drug Policy: The Case of the 1984 Hatch-Waxman Act

**DOI:** 10.1371/journal.pone.0351859

**Published:** 2026-07-07

**Authors:** Benjamin M. Blau, Joshua Lyman, Jason M. Smith, Ryan J. Whitby

**Affiliations:** 1 Department of Economics and Finance, Jon M. Huntsman School of Business, Utah State University, Logan, Utah, United States of America; 2 Department of Management Science and Engineering, Huang Engineering Center, Stanford University, Stanford, California, United States of America; The University of Tulsa, UNITED STATES OF AMERICA

## Abstract

This paper examines the effect of the 1984 Hatch-Waxman Act and its 2003 amendment – the Medicare Prescription Drug, Improvement, and Modernization Act – on the level of innovation in pharmaceutical firms. In part, the purpose of these policies was to balance the exclusivity rights of pioneer drug manufacturers with generic drug competition, which would allow for more consumer affordability. Using difference-in-difference regression analysis, we analyze how, relative to innovation in other industries, pharmaceutical innovation changes surrounding the implementation of the two laws. Results from several difference-in-difference tests show that, relative to non-pharmaceutical firms, research and development (R&D) in pharmaceutical firms markedly increased during the post-Hatch-Waxman period. We do not find, however, that the 2003 amendment directly influenced pharmaceutical innovation. If anything, the latter policy was associated with a reduction in innovation. These findings seem to suggest that the level of pharmaceutical innovation is highly sensitive to changes in policy that might change the incentives for firms to innovate.

## Introduction

The establishment of both economic and legal institutions plays a vital role in the development of markets and the efficient allocation of capital. There are few industries in which these institutions matter more to society’s standard of living than in the pharmaceutical industry as mortality rates for treatable diseases have declined dramatically. Over the last 100 years, pharmaceutical innovations like antibiotics and vaccines have decreased mortality rates from influenza and pneumonia by more than 85% while medical device innovations have lowered death rates from ulcers and heart disease by about 60% and 55%, respectively [1, 2]. Lichtenberg [3] shows that every $1 investment in pharmaceutical innovation results in a $3.65 reduction in hospital-care expenditures. Likewise, the production of every 100 new drugs is associated with a reduction in hospital visits of more than 16 days. Lichtenberg [4] shows that each new drug that receives Food and Drug Administration (FDA) approval saves over 11,000 life-years – producing a social rate of return of more than 40%.

Despite the social benefits associated with pharmaceutical innovation, the output of innovation has been decreasing dramatically over the last six or seven decades. Scannell et al. [5] show that, during the 1950s, a $1 billion investment in pharmaceutical research was associated with 40 new drugs. A similar investment during the first part of the 2000s only yields 0.7 new drugs. In fact, since 1950, the number of new pharmaceuticals has been cut in half approximately every nine years – thus highlighting the reduction in innovative productivity. Given the benefits associated with pharmaceutical innovation and the decline in its productivity, understanding the institutions that provide the framework for the pharmaceutical market has important implications. In this study, we attempt to examine how regulation targeting this industry influences the level of pharmaceutical innovation as measured by research and development (R&D) expenditures.

In light of the development of policies related to innovation, perhaps one of the most complex problems faced by economists and regulators is the balancing of exclusivity rights for new pioneer drug production and the promotion of generic drug competition to increase consumer affordability. On one hand, establishing temporary monopolies for new innovations provides the appropriate incentives for firms to develop and market new drugs. On the other hand, however, establishing competition in the generic drug market increases affordability and the public’s access to new drugs, which is ultimately how society benefits from pharmaceutical innovation. The Drug Price Competition and Patent Term Restoration Act – more commonly known as the Hatch-Waxman Act of 1984 – was a relatively modern piece of policy that attempted to balance exclusivity with affordability. Its objective was to bolster the generic drug market by reducing the requirements to replicate clinical tests for safety and efficacy for generic equivalent drugs that have the same basic pharmacokinetics and active ingredients as pioneer drugs. However, as part of the legislation, the 1984 Act also protected the patent rights of the pioneer drug producers by allowing for a 30-month legal stay on all potential generic infringements – a stay that could extend beyond the normal patent term. A loophole in the Act, however, allowed pioneer firms to obtain multiple 30-month stays by creating patents not only on active ingredients but also on packaging and other contents peripheral to the actual ingredients. This loophole received a great deal of criticism by policymakers and consumer advocates and resulted in an amendment to the Hatch-Waxman Act in 2003. The Medicare Prescription Drug, Improvement, and Modernization Act (MMA) of 2003 directly limited the use of more than one 30-month stay, among other things.

We have reason to believe that the Hatch-Waxman Act and its amendment may have had meaningful effects on the level of pharmaceutical innovation. At first glance, the bolstering of the generic drug market, which was the intent of Hatch-Waxman, might have reduced the level of pharmaceutical innovation as pioneer drug firms faced fewer barriers to entry and generic drug firms were not required to pay redundant R&D costs. The increased competition might have disincentivized pharmaceutical firms to innovate and R&D expenditures could have decreased. However, as discussed above, the loophole in Hatch-Waxman that allowed for multiple stays and the possibility of an even longer period of exclusivity may have had the opposite effect. Therefore, it is possible that the increased (temporary) monopoly power created by the loophole might have increased the level of pharmaceutical innovation.

The primary objective of this study is to examine how the 1984 Hatch-Waxman Act and its 2003 amendment (the MMA) influenced the level of pharmaceutical innovation, as measured by R&D expenditures. Specifically, we test two hypotheses using a difference-in-differences framework: (1) whether pharmaceutical R&D increased relative to non-pharmaceutical R&D following the Hatch-Waxman Act, consistent with the view that loopholes extending exclusivity rights incentivized innovation; and (2) whether the 2003 amendment, which closed these loopholes, led to a reduction in relative pharmaceutical R&D. To preview our findings, we document that pharmaceutical innovation increased significantly relative to non-pharmaceutical innovation following the Hatch-Waxman Act, while the MMA had little to no meaningful effect on relative pharmaceutical innovation.

The results from our tests contribute to the literature in two important ways. First, our findings show how sensitive pharmaceutical innovation is to changes in policy. Kesselheim [6] finds that from 1995 to 1999, the FDA approved 37.2 new drugs per year. From 2005 to 2009, that number dropped to 22.6 new drugs. In light of our results, perhaps the reduction in new drugs during the last 20 years can be explained by policy, which errs more on the side of affordability than on the side of exclusivity. Second, our findings more broadly contribute to the large literature that discusses the benefits associated with pharmaceutical innovation. As mentioned above, Lichtenberg [4] shows that the social rate of return on pharmaceutical innovation is approximately 40%. In the same study, Lichtenberg shows that for every $15 billion increase in pharmaceutical innovation, 1.6 million life-years are saved – an amount that is equivalent to about $27 billion in total economic contribution. Frech and Miller [7] show that pharmaceutical R&D directly affects life expectancy – particularly in older individuals. Combined with this extensive literature, the findings in this study seem to suggest that drug policy has important economic and societal consequences and new research is required to better understand the optimal drug policy that properly balances exclusivity with affordability.

The rest of this paper is organized as follows. The next section provides background on the two pieces of legislation and discusses the related literature. The Materials and Methods section describes the data, sample construction, and empirical approach. The Results section presents the empirical findings. The Discussion section interprets the results and considers policy implications. The final section offers concluding remarks.

## Background on the Hatch-Waxman Act and its amendment

The Drug Price Competition and Patent Term Restoration Act – more commonly termed as the Hatch-Waxman Act of 1984 – was introduced and passed through the U.S. Senate in June 1984. Less than three months later, the bill was passed by the House of Representatives and signed into law by President Ronald Reagan on September 24th, 1984. The objective of the bill was to provide a competitive framework for generic drug makers that would ultimately improve the affordability for consumers. Hatch-Waxman instituted changes to the Abbreviated New Drug Application (ANDA) that generic drug makers file to make generic equivalents to existing pioneer drugs as the original patent term was set to expire. The main implication from Hatch-Waxman was to allow ANDAs to be filed prior to the patent expiry and relax the restrictions by generic drug makers to provide the clinical trials for safety and efficacy that had already been performed by the pioneer drug makers when the New Drug Application (NDA) was first filed. Hatch-Waxman effectively reduced and eliminated repetitive R&D costs by allowing generic drugs to rely on previous clinical trials that had already determined the safety of the pioneer drug. Once the ANDA was approved by the FDA, the generic manufacturer was allowed to legally produce and market the generic equivalent to the existing pioneer drug. The expected implications of the policy were to improve the competitive environment of the generic drug market thus improving consumer affordability. However, Hatch-Waxman recognized the importance of exclusivity for pioneer drug makers. Without any exclusivity, the incentives to research and develop new drugs would decrease and could have harmful effects on the level of drug production, which could have adverse economic and societal effects. Therefore, Hatch-Waxman implemented a 30-month stay in response to any type of patent infringement. For instance, when an ANDA was filed that infringed on an existing NDA patent, the holder of the patent was given a 45-day period to file a formal complaint regarding the patent infringement. The FDA was then restricted from approving the ANDA by the Hatch-Waxman Act until the end of the 30-month stay. This important piece of the policy could allow the pioneer drug maker to extend the patent beyond its normal terms. Alternatively, however, the generic drug maker was not required to conduct a series of clinical tests to determine the safety and efficacy of the generic equivalent. Instead, the generic drug manufacturer was only required to wait for the 30-months before FDA approval of the ANDA, thus saving redundant costs associated with R&D.

Beyond providing an important discussion of the details behind the Hatch-Waxman Act, Avery [8] discusses an important loophole in the Act that allowed the pioneer drug company to obtain multiple 30-month stays. In particular, the pioneer drug maker could obtain multiple stays “through the use of so-called ‘sham’ patents, which claim features peripherally related to the patented drugs, such as metabolites, intermediates, and packaging features (pg 179-180).” Mahn [9] argued that this loophole became a strategic weapon of NDA patent holders to provide injunctive exclusivity – thus extending patent rights beyond normal patent terms. In this case, the loophole might have increased the incentive for drugmakers to invest in the research and development of new drugs. Our empirical tests that examine pharmaceutical innovation surrounding the Hatch-Waxman Act are an attempt to test this assertion.

Recognizing the implications of this loophole, policymakers set out to improve affordability by passing the Medicare Prescription Drug, Improvement, and Modernization Act of 2003 (MMA). The MMA amended some of Hatch-Waxman but specifically addressed the multiple 30-month stay loophole. In particular, the MMA addressed the ‘sham’ patent issue by revising the statute that required a 30-month stay for infringement on not only the initial patent but also on the subsequent ‘sham’ patents that were filed. The revision of this statute effectively restricted pioneer drug makers from obtaining multiple 30-month stays – thus ending the exclusivity rights 30-months after the normal patent expiry. While the MMA had many other legal implications for Hatch-Waxman, the closing of the ‘sham’ patent loophole seems most pressing for the tests that we conduct in this study. Avery [8] provides a more detailed discussion of both Hatch-Waxman and the MMA.

## Materials and methods

### Data sources

The data used throughout this analysis come from the Compustat North America Annual database, accessed through Wharton Research Data Services (WRDS). We note that Compustat provides strictly annual financial data; therefore, our analysis captures year-level variation in firm characteristics and R&D expenditures. We obtain annual firm data for the universe of firms for two periods. First, we gather data from 1982 to 1987 – the six-year period surrounding Hatch-Waxman. Second, we obtain data from 2001 to 2006 – the six-year period surrounding the MMA. All statistical analyses were performed using Stata (StataCorp, College Station, TX).

### Sample construction

Our analytical sample includes all firms in the Compustat database with non-missing data on R&D expenditures and total assets during the relevant sample periods. Pharmaceutical firms are identified using Standard Industrial Classification (SIC) codes between 2830 and 2836, which encompass firms in the industrial chemicals and pharmaceuticals sector. All remaining firms in the Compustat universe serve as the non-pharmaceutical control group. For the analysis surrounding the Hatch-Waxman Act, our sample spans 1982–1987, yielding 92 pharmaceutical firms and a broad cross-section of non-pharmaceutical firms. For the analysis surrounding the MMA, our sample spans 2001–2006, during which the number of pharmaceutical firms grew to 340. While Compustat access requires a subscription, it is widely available at academic institutions through WRDS, which supports reproducibility of our results.

### Variable definitions

Variables used in this analysis include: market capitalization (Mkt Cap) per firm per year; closing stock price (Price) of each firm per year; range-based volatility (Volatility), discussed in Alizadeh et al. [10], calculated as the difference between the natural log of the highest price and the natural log of the lowest price during a particular year; share turnover (Turn), or the ratio of trading volume scaled by shares outstanding for each firm in each year; the debt-to-equity ratio (D/E), measured as total liabilities scaled by total equity; return on equity (ROE), or the ratio of net income to total equity; annual research and development expenditures (R&D); and the ratio of R&D to total assets (R&D/Assets), which is the primary variable of interest throughout our study. Price data were obtained from the Center for Research in Security Prices (CRSP).

### Measuring innovation

We use R&D expenditures as our proxy for pharmaceutical innovation. This approach follows the knowledge production function framework established by Griliches [11], who demonstrated the conceptual link between R&D inputs and innovative output. Hall et al. [12] further document that R&D expenditure is strongly correlated with patent counts and citation-weighted patent output, supporting its validity as a measure of innovative effort. We acknowledge that R&D expenditures capture innovation inputs rather than outputs, and that alternative measures such as patent counts or new drug approvals also have well-known limitations. By scaling R&D by total assets (R&D/Assets), we isolate the level of innovative effort while controlling for firm size, allowing for meaningful comparisons across firms of different magnitudes.

### Empirical strategy

We employ a difference-in-differences research design to estimate the causal effect of the Hatch-Waxman Act and the MMA on pharmaceutical innovation. The treatment group consists of all firms classified under pharmaceutical SIC codes (2830–2836). The control group consists of all other firms in the Compustat universe. By using the broad universe of non-pharmaceutical firms as the control group, we capture the general economic trend in R&D spending, against which we measure the pharmaceutical-specific response to the legislation. This is a common approach in the applied economics literature for industry-level policy analysis. The treatment events are the passage of the Hatch-Waxman Act (September 1984) and, separately, the MMA (December 2003). A key identifying assumption of the difference-in-differences design is that, absent the legislation, pharmaceutical and non-pharmaceutical R&D would have followed parallel trends. We provide support for this assumption through a series of placebo-like tests described in the Results section.

Table 3 and subsequent tables report results for different specifications of the following regression:


$$\begin{array}{c} R\&D/{Assets}_{i,t}\;=\;\beta \text{0}\;+\;\beta \text{1}{Pharma}_{i}\;+\;\beta \text{2}{Event}_{t}\;+\;\beta \text{3}Pharma\times {Event}_{i,t}\;+\;\beta \text{4}{n(Cap)L}_{i,t}\;+\;\beta \text{5}{Price}_{i,t} \end{array}$$

$$\begin{array}{c} +\;\beta \text{6}{Turn}_{i,t}\;+\;\beta \text{7}{Volatility}_{i,t}\;+\;\beta \text{8}{D/E}_{i,t}\;+\;\beta \text{9}{ROE}_{i,t}+e_{i,t} \end{array}$$ (1)

The dependent variable is R&D/Assets, which measures the annual amount of R&D expenditures divided by the total assets of each firm in each year. Pharma is an indicator variable equal to one if firm i is considered a pharmaceutical firm (SIC code between 2830 and 2836) and zero otherwise. Event is an indicator variable equal to one for the period following the signing of the legislation and zero for the pre-legislation period. Pharma×Event is the interaction between these variables – the difference-in-differences estimator of primary interest. We include several control variables that might influence R&D expenditures: Ln(Cap), the natural log of the market capitalization; Price, the annual closing price obtained from CRSP; Volatility, the range-based volatility measure; Turn, share turnover; D/E, the debt-to-equity ratio; and ROE, return on equity. All specifications account for possible heteroskedasticity by using robust standard errors.

To assess potential multicollinearity among the control variables, we employ two approaches. First, we enter control variables one at a time across model specifications (Columns [2] through [7] of Tables 3–6), allowing the reader to observe the stability of the difference-in-differences estimator as additional covariates are included. The consistency of the Pharma×Event coefficient across specifications provides evidence against harmful multicollinearity. Second, we compute Variance Inflation Factors (VIFs) for the full specification and confirm that all VIFs fall below standard thresholds (VIF < 10), providing additional assurance that multicollinearity does not materially affect our estimates.

## Results

### Summary statistics

Table 1 reports statistics that summarize our first sample. In particular, we examine various firm statistics for our sample of pharmaceutical firms as well as our sample of non-pharmaceutical firms during the four-year period surrounding the Hatch-Waxman Act (1983–1986). As seen in the table, the average pharmaceutical firm had a market capitalization of $853 million, closing price of $16.46, volatility of 0.82, turnover of 0.686 million, current assets of $242 million, total assets of $460 million, current liabilities of $121 million, total liabilities of $204 million, a debt-to-equity ratio of 0.84, and an ROE of −0.33. We also note that R&D expenditures for the average pharmaceutical firm during the sample time period were approximately $36 million, which represented about 21% of total assets.

**Table 1:** Summary Statistics – The Hatch-Waxman Act of 1984.

	Pharmaceutical Firms			Non-Pharmaceutical Firms		
	Mean	Median	Std. Dev.	Mean	Median	Std. Dev.
	[1]	[2]	[3]	[4]	[5]	[6]
MktCap	852.52	41.86	2,187.85	371.03	38.82	1,753.26
Price	16.46	8.37	21.65	16.96	11.25	40.33
Volatility	0.82	0.76	0.45	0.67	0.55	0.48
Turn	685,597.16	557,203.56	582,722.91	14,887,012.83	370,308.49	883,092,095.00
Cur. Assets	242.44	8.71	610.91	221.27	18.75	1,117.95
Tot. Assets	460.08	13.45	1,236.85	1,556.59	54.79	7,164.71
Cur. Liab.	121.26	2.16	330.99	174.19	9.42	1,195.82
Tot. Liab.	203.96	3.26	606.20	1,229.61	26.20	6,486.67
D/E	0.84	0.49	4.97	2.26	1.17	17.89
ROE	−0.33	0.02	3.86	0.00	0.10	10.48
R&D	36.21	1.23	90.62	19.65	0.53	129.98
R&D/Assets	0.21	0.08	0.62	0.07	0.02	0.74

The table reports statistics that describe the sample of 92 pharmaceutical firms with SIC codes between 2830 and 2836 observed in 1984 – the year that the Hatch-Waxman Act was signed into law (September 24, 1984). MktCap is market capitalization for each firm in each year. Price is the closing stock price of each firm in each year. Volatility is a measure of range-based volatility discussed in Alizadeh et al. (2002) and is calculated as the difference between the natural log of the highest price and the natural of the lowest price during a particular year. Turn is the share turnover or the ratio of trading volume scaled by shares outstanding for each firm in each year. D/E is the debt-to-equity ratio measured as the amount of (annual) total liabilities scaled by (annual) total equity. ROE is the return on equity, or the ratio of net income to total equity. R&D is the amount of research and development expenditures for the year. The variable of interest throughout our study is R&D/Assets, which is the ratio of R&D to total assets.

The summary statistics for the average non-pharmaceutical firm are also reported in the table. Here, we find that the average non-pharmaceutical firm had market capitalization of $371 million, a share price of $16.96, volatility of 0.67, turnover of 14.9 million, current assets of $221 million, total assets of $1.6 billion, current liabilities of $174 million, total liabilities of $1.2 billion, a debt-to-equity ratio of 2.26, ROE of 0.00, R&D of $19.65 million, which represented about 7% of total assets.

Like Table 1, Table 2 reports the summary statistics for our second sample. For instance, the table provides the statistics for the sample of pharmaceutical firms and non-pharmaceutical firms during the four-year period surrounding the MMA (2002–2005). A few results are noteworthy when comparing the tables. First, the average pharmaceutical firm was much larger in terms of market capitalization during the second time period ($3.3 billion compared to $852 million). Similar inferences can be made when looking at non-pharmaceutical firms. We also find that both types of firms had more stock price volatility, more turnover, and more R&D during the latter time period.

**Table 2:** Summary Statistics – Medicare Prescription Drug, Improvement, and Modernization Act of 2003.

	Pharmaceutical Firms			Non-Pharmaceutical Firms		
	Mean	Median	Std. Dev.	Mean	Median	Std. Dev.
	[1]	[2]	[3]	[4]	[5]	[6]
MktCap	3,279.25	111.19	17,203.46	2,264.04	142.25	11,361.35
Price	10.18	3.99	15.18	34.35	12.72	1,159.54
Volatility	1.34	1.11	0.96	0.92	0.60	0.97
Turn	1,479,527.33	797,451.14	1,977,166.90	4,155,447.01	512,293.32	332,383,640.00
Cur. Assets	536.85	27.54	2,798.89	717.34	56.85	3,471.53
Tot. Assets	1,274.26	40.31	7,363.61	9,469.29	305.47	68,383.02
Cur. Liab.	286.20	6.50	1,730.24	601.78	28.88	4,268.85
Tot. Liab.	618.58	10.79	3,628.34	8,266.49	171.50	64,458.78
D/E	0.96	0.28	3.45	2.16	1.15	37.67
ROE	0.22	−0.29	26.86	−0.32	0.08	129.57
R&D	134.59	11.22	683.86	66.45	2.42	391.70
R&D/Assets	0.51	0.22	2.27	0.29	0.03	7.77

The table reports statistics that describe the sample of 340 pharmaceutical firms with SIC codes between 2830 and 2836 observed in 2003 – the year that the MMA was signed into law (December 8^th^, 2003). MktCap is market capitalization for each firm in each year. Price is the closing stock price of each firm in each year. Volatility is a measure of range-based volatility discussed in Alizadeh et al. (2002) and is calculated as the difference between the natural log of the highest price and the natural log of the lowest price during a particular year. Turn is the share turnover or the ratio of trading volume scaled by shares outstanding for each firm in each year. D/E is the debt-to-equity ratio measured as the amount of (annual) total liabilities scaled by (annual) total equity. ROE is the return on equity or the ratio of net income to total equity. R&D is the amount of research and development expenditures for the year. The variable of interest throughout our study is R&D/Assets, which is the ratio of R&D to total assets.

### Hatch-Waxman Act and pharmaceutical innovation

Column [1] of Table 3 is our most basic specification that only includes the indicator variables Pharma, Event, and their interaction (Pharma×Event). The positive and statistically significant coefficient in the first row of Column [1] of Table 3 indicates that pharmaceutical firms spend a larger proportion of their total assets on R&D compared to non-pharmaceutical firms. This result is also economically meaningful in that the coefficient of 0.0547 translates into more than 36 million dollars more spent on R&D at the average pharmaceutical firm compared to a non-pharmaceutical firm during the pre-event period. We do not find an increase in R&D for the average non-pharmaceutical firm around the Hatch-Waxman Act as indicated by the insignificant coefficient on our indicator variable Event. However, the positive (0.0739) and statistically significant (t-statistic = 2.93) coefficient on the interaction term Pharma×Event indicates that relative to non-pharmaceutical firms, pharmaceutical firms dramatically increased the amount they spent on R&D after the passing of the Hatch-Waxman Act. In economic terms, our difference-in-difference tests during the four-year period surrounding the Hatch-Waxman Act show that, relative to R&D in other industries, pharmaceutical R&D increased by about 7%, relative to total assets, during the two years following the passage of the Act. This result supports the idea that pharmaceutical firm spending is sensitive to changes in patent exclusivity.

**Table 3:** Difference-In-Difference Regressions: R&D Expenditures Surrounding Hatch-Waxman (1983–1986).

	[1]	[2]	[3]	[4]	[5]	[6]	[7]	[8]
Pharma	0.0547***	0.0584***	0.0554***	0.0540***	0.0505***	0.0552***	0.0552***	0.0515***
	(6.16)	(6.61)	(6.28)	(6.08)	(5.64)	(6.15)	(6.15)	(5.68)
Event	0.0023	0.0029	0.0021	0.0018	0.0045**	0.0024	0.0023	0.0039*
	(1.06)	(1.33)	(0.96)	(0.83)	(2.07)	(1.07)	(1.06)	(1.78)
Pharma × Event	0.0739***	0.0728***	0.0747***	0.0752***	0.0688***	0.0738***	0.0738***	0.0709***
	(2.93)	(2.90)	(2.97)	(2.96)	(2.76)	(2.91)	(2.92)	(2.92)
Ln(Cap)		−0.0055***						−0.0011*
		(−12.07)						(−1.70)
Price			−0.0006***					−0.0004***
			(−10.69)					(−5.61)
Turn				−1.0E-7				−2.0E-9
				(−0.64)				(−0.01)
Volatility					0.0514***			0.0456***
					(12.82)			(10.35)
D/E						−0.00004		−0.0005
						(−0.58)		(−0.50)
ROE							−0.00005	−0.0009
							(−0.16)	(−0.44)
R^2^	0.0346	0.0423	0.0454	0.0347	0.0599	0.0348	0.0348	0.0661
Robust SEs	Yes	Yes	Yes	Yes	Yes	Yes	Yes	Yes
N	8,870	8,870	8,870	8,709	8,870	8,864	8,861	8,700

The table reports the results from estimating the following equation using pooled firm-year data

*R&D/Assets*_*i,t*_ = β_0_ + β_*1*_*Pharma*_*i*_ + β_*2*_*Event*_*t*_ + β_*3*_*Pharma×Event*_*i,t*_ + β_*4*_*Ln(Cap)*_*i,t*_ + β_*5*_*Price*_*i,t*_ + β_*6*_*Turn*_*i,t*_ + β_*7*_*Volatility*_*i,t*_ + β_*8*_*D/E*_*i,t*_ + β_*9*_*ROE*_*i,t*_ + *e*_*i,t*_

The dependent variable is R&D/Assets for each stock *i* in each year *t.* The independent variables consist of the following: *Pharma* is an indicator variable equal to one if firm i is considered a pharmaceutical firm (SIC Code between 2830 and 2836) – zero otherwise. *Event* is a dummy variable equal to unity for the period following the signing of the Hatch-Waxman Act (1985–1986) – zero for years 1983 and 1984. *Pharma × Event* is the interaction between these variables. *Ln(Cap)* is the natural log of market capitalization. *Price* is the closing price obtained from CRSP. *Volatility* is a measure of range-based volatility discussed in Alizadeh et al. (2002) and is calculated as the difference between the natural log of the intraday high price and the natural log of the intraday low price. *D/E* is the debt-to-equity ratio measured as total liabilities scaled by total equity. *ROE* is the return on equity or the ratio of net income to total equity. We report t-statistics (in parentheses) from robust standard errors. *, **, and *** denote statistical significance at the 0.10, 0.05, and 0.01 levels, respectively.

Columns [2] through [7] of Table 3 adjust the baseline specification by including a variety of control variables. We include the control variables separately so that we can see which variables have the most impact on the relation between R&D expenditures and the Hatch-Waxman Act. In Column [2], we include a control variable for the size of the company, the natural log of market capitalization. In reality, we already control for the size of the firm partially when we construct our dependent variable as R&D expenditure relative to total assets. The negative and significant coefficient is indicative of the importance to control for size when examining R&D expenditures but the additional control for size does not impact the coefficient on Pharma×Event, which remains positive and significant (0.0728, t-statistic = 2.90). Column [3] includes the Price, which is negatively related to R&D/Assets but does not impact the coefficient on the interaction term. Columns [4], [6], and [7] include control variables for stock turnover, debt-to-equity, and return on equity, respectively and are not statistically different from zero. Column [5] includes Volatility, which has a coefficient of 0.0514 with a t-statistic equal to 12.82. There is a strong relation between higher stock return volatility and investing in R&D. However, this relation could stem from a variety of scenarios, so we want to take care when interpreting this coefficient. For the purposes of our study, it is sufficient to note that Pharma×Event remains positive and statistically significant, although the coefficient does decrease slightly. Column [8] is our complete specification that includes all of the control variables in the regression. We observe similar coefficients and statistical significance in the full specification, which alleviates possible concerns about multicollinearity between the control variables. Furthermore, finding very little variation in the size of the difference-in-difference estimator across columns seems to indicate that including various firm characteristics does not influence the effect of Hatch-Waxman on pharmaceutical innovation. In the end, when all of the control variables are included, we find a coefficient on Pharma×Event equal to 0.0709 with a t-statistic of 2.92, which indicates statistical significance at the 0.01 level. This result suggests that pharmaceutical firms increase their relative expenditures on R&D by more than 7% after the Hatch-Waxman Act. A 7% increase by the average firm would equate to more than 47 million dollars (holding total assets constant). It is also worth noting that all of our specifications account for possible heteroskedasticity by using robust standard errors.

To examine the robustness of our results, we extend the period around the Hatch-Waxman Act from four years (1983–1986) to six years (1982–1987). Table 4 reports the results from the analysis that is analogous to Table 3 but uses the longer event window. Column [1] is our base specification that only includes the indicator variables for Pharma, Event, and Pharma×Event. Columns [2] through [7] each include an additional control variable that could potentially impact R&D expenditures. Column [8] includes the full specification with all of the indicator variables and all of the control variables. Since results for the six-year period are very similar to results for the four-year period, for brevity, we focus our attention on the full specification in Column [8]. Firms in the pharmaceutical industry spend more on R&D compared to other industries during the pre-event period as evidenced by the positive and statistically significant coefficient on Pharma (0.0620, t-statistic = 6.06). Although the coefficient on the interaction term (Pharma×Event) is smaller than in Table 3, 0.0524 compared to 0.0709, the estimate using the longer event window is still statistically significant with a t-statistic of 2.83 and economically meaningful with an increase of almost 35 million dollars for the average firm. Both the sign and significance of control variable coefficients are similar in Tables 3 and 4.

**Table 4:** Difference-In-Difference Regressions: R&D Expenditures Surrounding Hatch-Waxman (1982–1987).

	[1]	[2]	[3]	[4]	[5]	[6]	[7]	[8]
Pharma	0.0640***	0.0681***	0.0656***	0.0633***	0.06011***	0.0646***	0.0646***	0.0620***
	(6.43)	(6.87)	(6.65)	(6.37)	(5.97)	(6.43)	(6.43)	(6.06)
Event	0.0022	0.0032*	0.0015	0.0018	0.0021	0.0022	0.0023	0.0018
	(1.28)	(1.83)	(0.87)	(1.00)	(1.21)	(1.28)	(1.28)	(1.02)
Pharma × Event	0.0649***	0.0631***	0.0640***	0.0602***	0.0582***	0.0644***	0.0644***	0.0524**
	(3.23)	(3.17)	(3.21)	(3.10)	(2.93)	(3.19)	(3.20)	(2.83)
Ln(Cap)		−0.0053***						−0.0017***
		(−14.12)						(−3.72)
Price			−0.0006***					−0.0003***
			(−12.57)					(−5.54)
Turn				−3.0E-7				−3.0E-7
				(−1.09)				(−0.83)
Volatility					0.0486***			0.0446***
					(13.49)			(11.92)
D/E						−0.00006		−0.0007
						(−0.70)		(−0.68)
ROE							−0.00009	−0.0012
							(−0.26)	(−0.58)
R^2^	0.0403	0.0481	0.0498	0.0384	0.0649	0.0405	0.0405	0.0712
Robust SEs	Yes	Yes	Yes	Yes	Yes	Yes	Yes	Yes
N	13,047	13,047	13,047	12,797	13,047	13,038	13,033	12,783

The table reports the results from estimating the following equation using pooled firm-year data

R&D/Assets_*i,t*_ = β_*0*_ *+ β*_*1*_*Pharma*_*i*_ + β_*2*_*Event*_*t*_ + β_*3*_*Pharma*×*Event*_*i,t*_ + β_*4*_*Ln(Cap)*_*i,t*_ + β_*5*_*Price*_*i,t*_ + β_*6*_*Turn*_*i,t*_ + β_*7*_*Volatility*_*i,t*_ + β_*8*_*D/E*_*i,t*_ + β_*9*_*ROE*_*i,t*_ *+ e*_*i,t*_

The dependent variable is R&D/Assets for each stock *i* in each year *t.* The independent variables consist of the following: *Pharma* is an indicator variable equal to one if firm i is considered a pharmaceutical firm (SIC Code between 2830 and 2836) – zero otherwise. *Event* is a dummy variable equal to unity for the period following the signing of the Hatch-Waxman Act (1985–1987) – zero for years 1982–1984. *Pharma × Event* is the interaction between these variables. *Ln(Cap)* is the natural log of market capitalization. *Price* is the closing price obtained from CRSP. *Volatility* is a measure of range-based volatility discussed in Alizadeh et al. (2002) and is calculated as the difference between the natural log of the intraday high price and the natural log of the intraday low price. *D/E* is the debt-to-equity ratio measured as total liabilities scaled by total equity. *ROE* is the return on equity or the ratio of net income to total equity. We report t-statistics (in parentheses) from robust standard errors. *, **, and *** denote statistical significance at the 0.10, 0.05, and 0.01 levels, respectively.

### The medicare prescription drug, improvement, and modernization act and pharmaceutical innovation

While the exogenous shock of the Hatch-Waxman Act helps us to isolate the relation between patent exclusivity and R&D expenditures in the pharmaceutical industry, the reversal of some of the provisions in the Hatch-Waxman Act by the MMA in 2003 is a rare opportunity to validate the nature of the relation demonstrated in our previous findings. Thus, we duplicate the analysis from Tables 3 and 4 performed around the Hatch-Waxman Act in Tables 5 and 6 but use the MMA legislation of 2003 as our event.

**Table 5:** Difference-In-Difference Regressions: R&D Expenditures Surrounding MMA (2002–2005).

	[1]	[2]	[3]	[4]	[5]	[6]	[7]	[8]
Pharma	0.1893***	0.1901***	0.1863***	0.1884***	0.1764***	0.1901***	0.1968***	0.1856***
	(15.10)	(15.54)	(15.19)	(15.04)	(14.55)	(15.08)	(13.35)	(12.37)
Event	0.0040	0.0095	0.0113	0.0040	0.0193***	0.0041	0.0043	0.0173***
	(0.60)	(1.27)	(1.56)	(0.60)	(2.59)	(0.61)	(0.67)	(2.69)
Pharma × Event	−0.0178	−0.0216	−0.0243	−0.0165	−0.0143	−0.0188	−0.0271	−0.0282
	(−1.08)	(−1.32)	(−1.48)	(−1.00)	(−0.89)	(−1.14)	(−1.46)	(−1.54)
Ln(Cap)		−0.0180***						−0.0086***
		(−6.16)						(−2.73)
Price			−0.0018***					−0.0007***
			(−10.04)					(−6.56)
Turn				−1.0E-6***				−2.0E-6***
				(−5.07)				(−3.01)
Volatility					0.0569***			0.0370***
					(8.82)			(7.08)
D/E						-3E-8		−0.0023***
						(−0.00)		(−3.16)
ROE							−0.0084	−0.0136***
							(−1.48)	(−3.12)
R^2^	0.0264	0.0370	0.0356	0.0261	0.037	0.0263	0.1121	0.1802
Robust SEs	Yes	Yes	Yes	Yes	Yes	Yes	Yes	Yes
N	11,974	11,974	11,974	11,878	11,974	11,943	11,941	11,845

The table reports the results from estimating the following equation using pooled firm-year data

R&D/Assets_*i,t*_ = β_*0*_ *+ β*_*1*_*Pharma*_*i*_ + β_*2*_*Event*_*t*_ + β_*3*_*Pharma*×*Event*_*i,t*_ + β_*4*_*Ln(Cap)*_*i,t*_ + β_*5*_*Price*_*i,t*_ + β_*6*_*Turn*_*i,t*_ + β_*7*_*Volatility*_*i,t*_ + β_*8*_*D/E*_*i,t*_ + β_*9*_*ROE*_*i,t*_ *+ e*_*i,t*_

The dependent variable is R&D/Assets for each stock *i* in each year *t.* The independent variables consist of the following: *Pharma* is an indicator variable equal to one if firm i is considered a pharmaceutical firm (SIC Code between 2830 and 2836) – zero otherwise. *Event* is a dummy variable equal to unity for the period following the signing of the Medicare Modernization Act (2004–2005) – zero for years 2002 and 2003. *Pharma × Event* is the interaction between these variables. *Ln(Cap)* is the natural log of market capitalization. *Price* is the closing price obtained from CRSP. *Volatility* is a measure of range-based volatility discussed in Alizadeh et al. (2002) and is calculated as the difference between the natural log of the intraday high price and the natural log of the intraday low price. *D/E* is the debt-to-equity ratio measured as total liabilities scaled by total equity. *ROE* is the return on equity or the ratio of net income to total equity. We report t-statistics (in parentheses) from robust standard errors. *, **, and *** denote statistical significance at the 0.10, 0.05, and 0.01 levels, respectively.

**Table 6:** Difference-In-Difference Regressions: R&D Expenditures Surrounding MMA 2003 (2001–2006).

	[1]	[2]	[3]	[4]	[5]	[6]	[7]	[8]
Pharma	0.1971***	0.1980***	0.1957***	0.1968***	0.1851***	0.1981	0.2022***	0.1914***
	(18.96)	(19.48)	(19.23)	(18.90)	(18.02)	(18.96)	(17.60)	(16.43)
Event	−0.0008	0.0058	0.0073	−0.0008	0.0222***	−0.0007	0.0001	0.0219***
	(−0.15)	(1.02)	(1.34)	(−0.16)	(3.11)	(−0.14)	(0.02)	(3.29)
Pharma × Event	−0.0004	−0.0049	−0.0089	0.0003	−0.0001	−0.0014	−0.0068	−0.0116
	(−0.02)	(−0.31)	(−0.57)	(0.02)	(−0.01)	(−0.09)	(−0.41)	(−0.70)
Ln(Cap)		−0.0053***						−0.0074***
		(−14.12)						(3.43)
Price			−0.0017***					−0.0006***
			(−12.30)					(−7.16)
Turn				−2.0E-7***				−3.0E-6***
				(−4.63)				(−3.75)
Volatility					0.0680***			0.0504***
					(7.02)			(5.06)
D/E						-1E-5		−0.0021***
						(−0.43)		(−2.79)
ROE							−0.0080	−0.0127***
							(−1.46)	(−2.75)
R^2^	0.0362	0.0471	0.0462	0.0361	0.0533	0.0363	0.0973	0.1537
Robust SEs	Yes	Yes	Yes	Yes	Yes	Yes	Yes	Yes
N	18,121	18,121	18,121	17,974	18,121	18,071	18,069	17,922

The table reports the results from estimating the following equation using pooled firm-year data

R&D/Assets_*i,t*_ = β_*0*_ *+ β*_*1*_*Pharma*_*i*_ + β_*2*_*Event*_*t*_ + β_*3*_*Pharma*×*Event*_*i,t*_ + β_*4*_*Ln(Cap)*_*i,t*_ + β_*5*_*Price*_*i,t*_ + β_*6*_*Turn*_*i,t*_ + β_*7*_*Volatility*_*i,t*_ + β_*8*_*D/E*_*i,t*_ + β_*9*_*ROE*_*i,t*_ *+ e*_*i,t*_

The dependent variable is R&D/Assets for each stock *i* in each year *t.* The independent variables consist of the following: *Pharma* is an indicator variable equal to one if firm i is considered a pharmaceutical firm (SIC Code between 2830 and 2836) – zero otherwise. *Event* is a dummy variable equal to unity for the period following the signing of the Medicare Modernization Act (2004–2006) – zero for years 2001–2003. *Pharma × Event* is the interaction between these variables. *Ln(Cap)* is the natural log of market capitalization. *Price* is the closing price obtained from CRSP. *Volatility* is a measure of range-based volatility discussed in Alizadeh et al. (2002) and is calculated as the difference between the natural log of the intraday high price and the natural log of the intraday low price. *D/E* is the debt-to-equity ratio measured as total liabilities scaled by total equity. *ROE* is the return on equity or the ratio of net income to total equity. We report t-statistics (in parentheses) from robust standard errors. *, **, and *** denote statistical significance at the 0.10, 0.05, and 0.01 levels, respectively.

Table 5 reports results for the four-year window that spans from 2002 to 2005. Variable definitions are the same as previously described. Table 5 follows the format of the previous tables with the base specification detailed in Column [1], the full specification that includes all the control variables reported in Column [8], and columns [2] through [7] used to report the addition of individual control variables to the base specification. Although the format of Table 5 is similar to previous tables, the results are quite different. It is important to remember that the MMA occurred two decades after the Hatch-Waxman Act and the number of firms listed as members of the pharmaceutical industry grew from 92 to 340. Not only did the number of firms increase but the size of those firms also increased with average total assets increasing from $663 million to $2.16 billion and market capitalization increasing from $923 million to $5.385 billion. Moreover, the amount that pharmaceutical firms spent on R&D more than quadrupled. This is also evident in the regression results reported in Table 5 where the coefficient on the indicator variable for Pharma is 0.1893. During the pre-Hatch-Waxman period (in Table 3), the average pharmaceutical firm spent about 6% of its total assets on R&D. By 2003, the average pharmaceutical firm spent almost 19% of its assets on R&D each year. Given the steady increase of R&D expenditures by pharmaceutical firms through time, one might expect this trend to continue after the passing of the MMA legislation. However, we find that the additional restriction on the exclusivity of pharmaceutical products instituted by the MMA legislation resulted in a decrease in relative R&D expenditures for pharmaceutical firms. Row 3 of Table 5 reports the results for the change in R&D/Assets around the MMA legislation. Coefficients range from −0.0282 to −0.0143, with t-statistics of −1.54 and −0.89, respectively. Although none of the coefficients on the interaction terms are statistically different from zero at standard statistical cutoff levels, they are all negative and quite consistent. These findings are consistent with the idea that patent exclusivity impacts the expected profitability of R&D investments and that firms adjust their R&D expenditures according to changes in the legislative environment. We note that in the full specification, the interaction term produces a coefficient of −0.0282 with a t-statistic of −1.54. While not statistically significant at the 0.10 level, the t-statistic is quite large – with a p-value of 0.1236. Therefore, we find some weak evidence that, in response to MMA, pharmaceutical innovation decreased relative to non-pharmaceutical innovation.

Results for our difference-in-difference analysis around the 2003 MMA legislation using a six-year window are reported in Table 6. Results are similar to Table 5, so for brevity, we will focus our attention on the full specification and other differences worth noting. First, it is worth noting that the trend of increasing R&D expenditures in the pharmaceutical industry is quite strong. Adding one additional year to the end of our sample increased the percentage of assets spent on R&D by pharmaceutical companies by almost 1 percent (in most specifications). It is also worth noting that the longer event window and the general trend toward increased R&D expenditures start to limit our event window tests. Thus, the indicator variable around the MMA event in Table 6 is effectively zero with very small coefficients and no statistical significance. However, it is important to remember that zero growth in R&D expenditures over a six-year period is quite rare given the historic growth rates. Furthermore, it is also worth noting that in contrast to the results around the Hatch-Waxman Act, all of the control variables are statistically significant in our analysis around the MMA legislation. R&D/Assets is negatively related to Ln(Cap), Price, Turn, D/E, and ROE. The only control variable that is positively related to R&D expenditures is Volatility, which is consistent with results from previous tables. Combined, the results in this subsection suggest that, in response to the MMA, pharmaceutical innovation – relative to non-pharmaceutical innovation – does not meaningfully change. While Table 5 provides weak evidence, at best, that pharmaceutical innovation vis-à-vis non-pharmaceutical innovation decreases in response to the MMA, Table 6 does not provide any evidence that relative pharmaceutical innovation changes during the post-event period.

### Robustness tests

**Fig 1:**
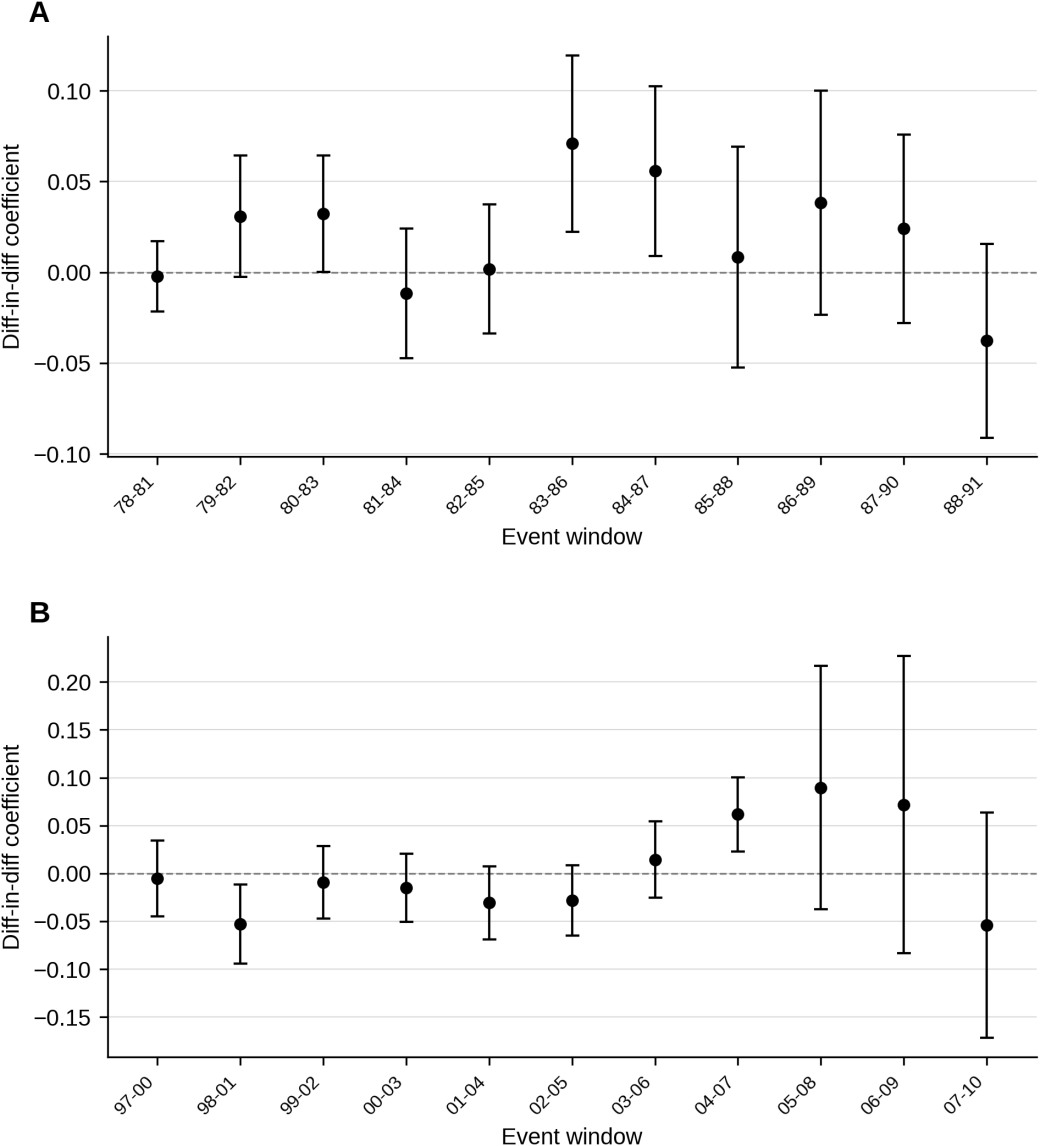
Difference-in-difference coefficients across event windows surrounding Hatch-Waxman and the MMA. The figure shows a replication of the multivariate, difference-in-difference regression analysis for various event windows. (A) shows the results for 10 (four-year) event windows surrounding the Hatch-Waxman Act, with the true event window of 1983 to 1986. (B) shows the results for 10 (four-year) event windows surrounding the Drug Modernization Act, with the true event window of 2002 to 2005. The difference-in-difference coefficients are obtained from estimating the full specifications that include all of the control variables. The point estimates are surrounded by two standard error upper bands and two standard error lower bands.

Thus far, we have documented that pharmaceutical innovation increases (relative to non-pharmaceutical innovation) during the period after Hatch-Waxman but not after the MMA. Of course, our analysis is attempting to make causal inferences about how the legislation affects the level of innovation. Admittedly, however, observing significant difference-in-difference estimates is not tantamount to identifying a causal link, which is nearly impossible given that the motives behind those making decisions about R&D expenditures are unobserved. Recognizing these limitations in our tests, this section replicates our analysis in the previous two subsections but instead of analyzing only the period surrounding the two pieces of legislation, we conduct placebo-like tests to give us more confidence in the conclusions we draw from our earlier findings. In particular, we re-estimate the full specification of Eq (1) for 20 different event windows that are outside of Hatch-Waxman and the MMA event periods. Fig 1 reports the results from the analysis when looking at the various four-year event windows. In the figure, we plot the difference-in-difference point estimates along with confidence bands (two standard error upper bands and two standard error lower bands) for the various placebo-like event windows. For instance, in the first event window, which extends from 1978 to 1981, the indicator variable POST represents the two-year period from 1980 to 1981 – zero otherwise. Similarly, in the last event window from 1988 to 1991, the POST variable captures the two-year period from 1990 to 1991. In the top panel of Fig 1, we show the results for five event windows before Hatch-Waxman and five event windows after Hatch-Waxman. Here, we observe that the difference-in-difference point estimate for the event window from 1983 to 1986, which is the same event window reported in Table 3, is 0.0709 and, according to the confidence bands, the estimate is statistically different from zero. These results have been already shown in Table 3. We note that in the other 10 (four-year) event windows, the only other event window that shows a positive and significant difference-in-difference estimate is the window from 1984 to 1987, which captures some of the post-Hatch-Waxman Act time period. In the other nine placebo-like tests, we do not find estimates that are meaningfully different from zero. This exercise seems to suggest that indeed the Hatch-Waxman Act influenced the level of pharmaceutical innovation and the increase in such innovation was not driven by some other confounding event during the years surrounding Hatch-Waxman.

**Fig 2:**
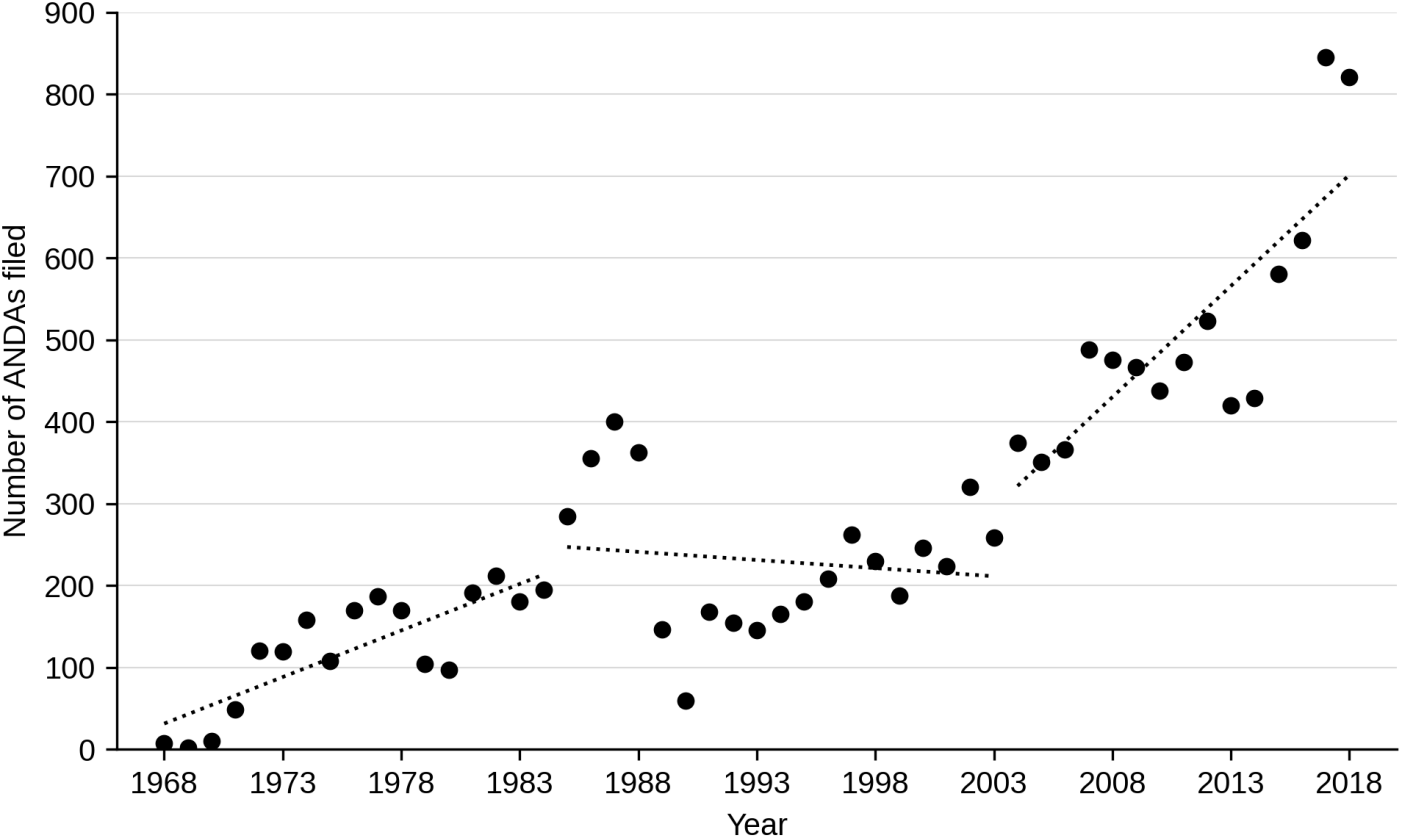
Annual Abbreviated New Drug Application (ANDA) filings, 1968–2018. The figure shows annual trends for Abbreviated New Drug Applications (ANDAs) for the period prior to the Hatch-Waxman Act (1968-1984), the period between the Hatch-Waxman Act and the Drug Modernization Act (1985-2003), and the period after the Drug Modernization Act (2004-2018).

Fig 2 provides an initial descriptive look at the effects of the Hatch-Waxman loophole. After hand collecting data on the number of ANDAs, which are filed by pharmaceutical firms when making a generic equivalent to an existing pioneer drug, we plot the filings for the period prior to the Hatch-Waxman Act (1968–1984) and the period following the Act (1985–2003). Of course, the intention of the legislation was to increase affordability by generating competition through a stronger, more robust generic drug market. However, as seen in the figure, the trend in the number of ANDAs decreased instead of increased during the period following the Hatch-Waxman Act. It appears that the loopholes in the legislation resulted in less generic drug production. Given these findings, the increase in pharmaceutical innovation (rather than a decrease) in response to the Hatch-Waxman Act is consistent with our regression results. When the loopholes were closed by the passage of the 2003 MMA, the figure shows that the trend in ANDAs increases dramatically.

In the bottom panel of Fig 1, we conduct the same type of robustness tests for 10 (four-year) event windows surrounding the MMA. Like in Table 5, the difference-in-difference estimate for the event window that extends from 2002 to 2005 is −0.0282 but is not reliably different from zero. We do find some evidence that, in the event window from 2004 to 2007, the difference-in-difference point estimate is positive and reliably different from zero. This increase in pharmaceutical (compared to non-pharmaceutical) innovation is likely due to the Medicaid Part D expansion that went into effect beginning in 2006. With expanded spending on Medicaid, our results in the bottom panel of Fig 1 are consistent with the idea that pharmaceutical firms had greater incentive to allocate capital to research and development. While the difference-in-difference point estimates increase again for the event windows extending from 2005 to 2008 and from 2006 to 2009, the standard errors blow up, which is likely due to the effects of the financial crisis. Therefore, we cannot make any meaningful conclusions about how pharmaceutical innovation responded during the later period covering the financial crisis.

## Discussion

Combined, the results from our analyses highlight the sensitivity of pharmaceutical innovation to incentives inherent in policy. Our difference-in-difference tests indicate that pharmaceutical R&D (as a percent of total assets) increased significantly, relative to non-pharmaceutical R&D, during the period immediately following the passage of the Hatch-Waxman Act. These results are robust to various time windows surrounding the Act and the inclusion of a variety of firm characteristics. To the extent that the loopholes in Hatch-Waxman indeed extended the exclusivity rights of pioneer drug manufacturers thus discouraging generic drug production, observing an increase in pharmaceutical innovation is consistent with our expectations.

When replicating our analysis around the 2003 amendment, we do not find that pharmaceutical R&D increased during the post-MMA period. In fact, if anything, pharmaceutical R&D, compared to non-pharmaceutical R&D, decreased during the post-MMA period. Comparing the results between the two event studies, our findings indicate that while the Hatch-Waxman Act enhanced pharmaceutical innovation, the MMA did not. Combined, these findings highlight the sensitivity of pharmaceutical innovation to changes in exclusivity rights. Even temporary monopoly power plays a crucial role in establishing the appropriate incentives for pharmaceutical firms to innovate.

At a very minimum, our findings indicate that policies that limit the profitability of pioneer drugs can adversely affect the level of pharmaceutical innovation, which, under certain conditions, can be harmful to society. Admittedly, unaffordable existing drugs can also have harmful effects on society. Therefore, finding the optimal balance between exclusivity and affordability is essential when developing optimal drug policies. Given the societal benefits associated with pharmaceutical innovation, at a very minimum, policymakers ought to consider this sensitivity when proposing legislation that could affect the level of innovation.

## Conclusion

Prior research documents the societal and economic benefits associated with pharmaceutical innovation. However, these benefits are not brought about by the innovation itself, but instead by the consumption of the innovation. For instance, while the presence of new pharmaceuticals has shown to increase life expectancies and reduce aggregate health care costs, it is the consumption of these pharmaceuticals that generate societal and economic benefits. Therefore, policymakers should not only be concerned about creating the institutional framework that promotes innovation, but a framework should also be established that encourages affordability to maximize the consumption of the innovation. The Hatch-Waxman Act of 1984 was an attempt to balance the exclusivity rights of pioneer drug makers – to promote innovation – and the development of a competitive generic drug market – to increase consumer affordability. Loopholes in Hatch-Waxman led to injunctive exclusivity by allowing for multiple monopolistic stays for certain pharmaceuticals that extended beyond normal patent terms. As a result, our difference-in-difference tests indicate that pharmaceutical R&D (as a percent of total assets) increased significantly, relative to non-pharmaceutical R&D, during the period immediately following the passage of Hatch-Waxman. These results are robust to various time windows surrounding the Act and the inclusion of a variety of firm characteristics.

Due to the criticism about the loopholes in Hatch-Waxman, policymakers amended the Act in 2003 by passing the MMA. This amendment restricted the number of monopolistic stays to only one. This reduction in the exclusivity term might also affect the level of innovation in pharmaceutical firms. When we replicate the difference-in-difference tests surrounding the MMA, we find some weak evidence that, relative to non-pharmaceutical innovation, pharmaceutical R&D decreases. However, these results are not very strong nor are the results robust to various time windows surrounding MMA. We are left to conclude that while Hatch-Waxman appeared to increase pharmaceutical innovation, the amendment to Hatch-Waxman had little to no effect on the level of innovation in the pharmaceutical industry.

Our findings contribute to the literature by highlighting the importance of both economic and legal institutions in the market for innovation. There are likely fewer economic problems that have a more profound effect than finding the optimal balance between exclusivity for pioneer drug makers and the development of a robust generic drug market. Both theoretical and additional empirical research in this area is needed to better understand the important tradeoffs between exclusivity and affordability.
